# The promising therapeutic effects of metformin on metabolic reprogramming of cancer-associated fibroblasts in solid tumors

**DOI:** 10.1186/s11658-022-00356-2

**Published:** 2022-07-22

**Authors:** Samaneh Mostafavi, Hamidreza Zalpoor, Zuhair Mohammad Hassan

**Affiliations:** 1 Department of Immunology, Faculty of Medical Sciences, Tarbiat Modares University, 14115-154, Tehran, Iran; 2 Shiraz Neuroscience Research Center, Shiraz University of Medical Sciences, Shiraz, Iran; 3 Network of Immunity in Infection, Malignancy and Autoimmunity (NIIMA), Universal Scientific Education and Research Network (USERN), Tehran, Iran

**Keywords:** Tumor microenvironment, Myofibroblasts, Cancer-associated fibroblasts, Metformin, Lactic acid, Stromal cells

## Abstract

Tumor-infiltrated lymphocytes are exposed to many toxic metabolites and molecules in the tumor microenvironment (TME) that suppress their anti-tumor activity. Toxic metabolites, such as lactate and ketone bodies, are produced mainly by catabolic cancer-associated fibroblasts (CAFs) to feed anabolic cancer cells. These catabolic and anabolic cells make a metabolic compartment through which high-energy metabolites like lactate can be transferred via the monocarboxylate transporter channel 4. Moreover, a decrease in molecules, including caveolin-1, has been reported to cause deep metabolic changes in normal fibroblasts toward myofibroblast differentiation. In this context, metformin is a promising drug in cancer therapy due to its effect on oncogenic signal transduction pathways, leading to the inhibition of tumor proliferation and downregulation of key oncometabolites like lactate and succinate. The cross-feeding and metabolic coupling of CAFs and tumor cells are also affected by metformin. Therefore, the importance of metabolic reprogramming of stromal cells and also the pivotal effects of metformin on TME and oncometabolites signaling pathways have been reviewed in this study.

## Introduction

**Fig. 1:**
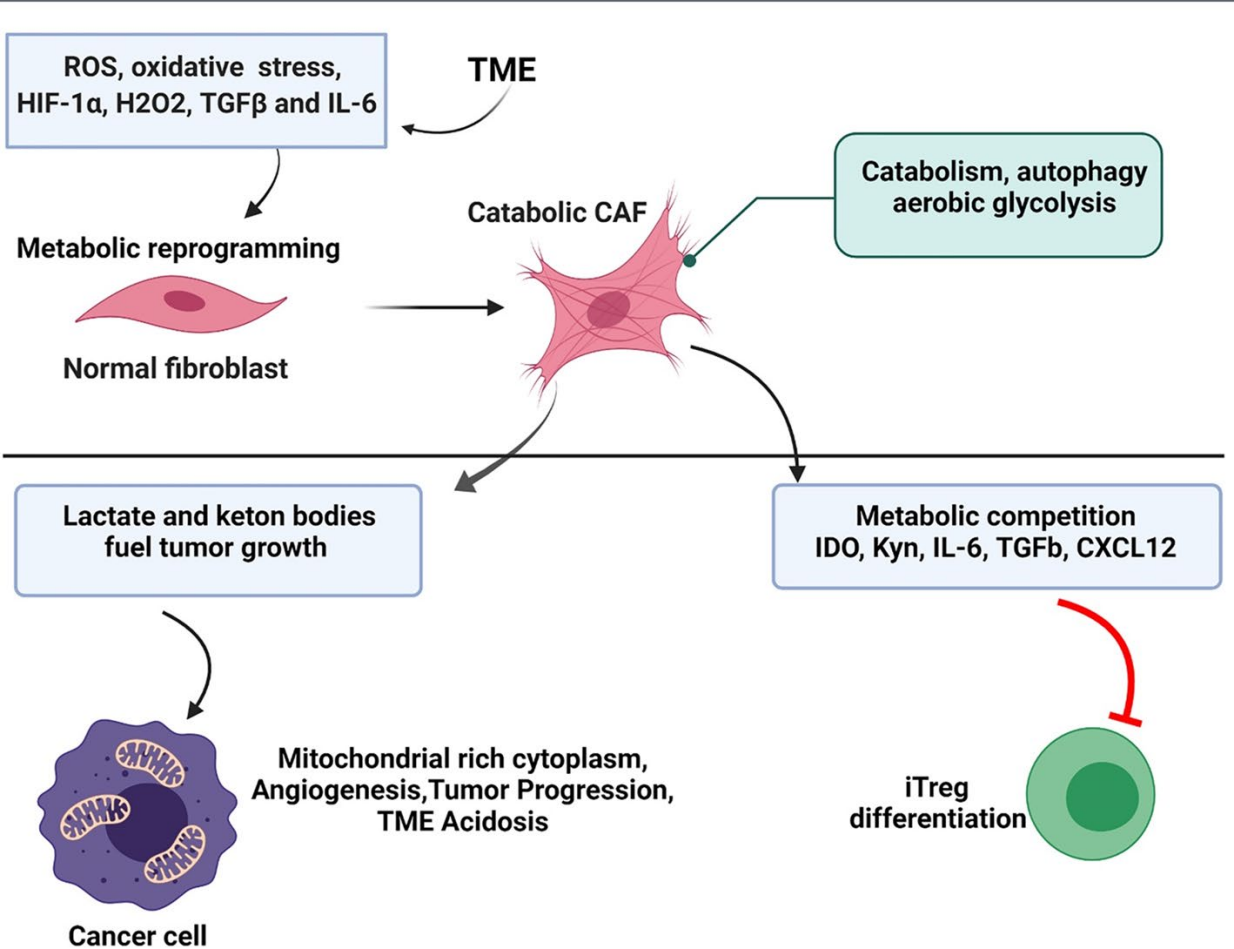
Effects of CAF differentiation in the TME. Toxic TME drives metabolic reprogramming in the normal fibroblast towards the catabolic CAF. These cells are highly glycolytic, providing lactate and ketone bodies to fuel tumor growth and progression. The aberrant secretome of CAFs (also referred to as “cancer cell secretome”) contributes to metabolic stress in the TME and also inhibits the function of effector T cells and enables iTreg differentiation.* CXCL12* C-X-C motif chemokine 12, * IDO* indoleamine 2,3-dioxygenase,* IL* interleukin, *iTreg* induced regulatory T cells,* TGF* transforming growth factor, *TME* tumor microenvironment; for other abbreviations, see Abbreviation section

Among stromal cells, the most important and challenging is the myofibroblast or cancer-associated fibroblast (CAF), which not only provides metabolic support to cancer cells, but their secretome also makes tumors resistant to therapy (Fig. 1) [1]. CAFs are a dynamic group of cells, capable of differentiating into various subtypes with different phenotypes during their activation phase (Table 1) [2]. Understanding the specific characteristics of each subset enables healthcare providers to select an appropriate therapy [3]. Fibroblasts are non-epithelial cells (E-cadherin^−^, cytokeratin^−^), non-endothelial (CD31^−^), and non-immune cells (CD45^−^). They are differentiated from local mesenchymal stem cells (MSC) or mesenchymal cells that are recruited from bone marrow [4, 5]. Fibroblast heterogeneity has been shown to be correlated with tumor metastasis and invasion in breast cancer (Table 1) [6]. For example, CAF-S1 and CAF-S4 are responsible for axillary lymph nodes metastasis, and their function relies on the transforming growth factor beta (TGF-β), chemokine (C-X-C motif) ligand (CXCL)12, and NOTCH signaling pathways [7]. Metabolism and transcriptional activity in quiescent or resting fibroblasts are low, and such fibroblasts do not migrate between tissues. However, when fibroblasts are activated by different molecules of the tumor microenvironment (TME), such as cancer-derived reactive oxygen species (ROS) and cytokines derived from malignant and immune cells, such as TGF-β, platelet-derived growth factor (PDGF), and fibroblast growth factor 2 (FGF2), they differentiate into CAFs [8]. Oncometabolites, such as lactate, produced by pancreatic cancer cells, also affect CAF differentiation [9]. It has also been demonstrated that 2-hydroxyglutarate, which is an important oncometabolite in TME, not only suppresses anti-tumor immune functions but is also taken up by primary fibroblasts, which in turn increases proliferation and differentiation to CAFs [10, 11]. Myofibroblasts are recognized by alpha-smooth muscle actin (aSMA) or fibroblast activation protein (FAP) [8, 12].

**Table 1:** The heterogeneity of cancer-associated fibroblasts

Category of CAFs	References	Function in TME
S1–S4	[6]	CAF-S1 is the main functioning subtype in triple negative breast cancer cells, providing an immunosuppressive TME CAF-S2 is abundant in LumA breast cancer CAF-S3 is distributed symmetrically in each type of breast cancer CAF-S4 is more frequent in HER2-positive tumors and is involved in cytoskeleton rearrangement and aerobic metabolism
F1–F4	[1]	F1 prevents tumor progression and keeps it under control F2 promotes cancer development and might assist in survival and metastasis F3 modulates the immune cells, and assists in tumor latency, angiogenesis, and invasion F4 involved in ECM interactions and signaling
vCAF mCAF cCAF dCAF	[177]	Occupies the tumor core, angiogenesis, and vascular development Constructing the invasive front of tumors, and ECM remodeling Also known as highly proliferative vCAFs Expresses genes related to cancer stem cells
Extracellular matrix		

*TME* Tumor microenvironment, *CAF* cancer-associated fibroblast,* vCAF* vascular CAF,* mCAF* matrix CAF,* cCAF* cycling CAF,* dCAF* developmental CAF,* ECM* extracellular matrix

Tumor cells are highly proliferative and need energy for their rapid growth. Therefore, their ability to take up glucose and high-energy metabolites is likely to be very crucial [13]. To this end, cancer cells rewire their metabolism (also known as metabolic reprogramming) to overcome the high demand for energy together with metabolic symbiosis with stromal cells [14]. That metabolic cross-talk between cancer cells and their stroma provides energy for the rapid growth of cancer cells is now well recognized and considered to be the main cause of cancer progression, invasion, and chemotherapy resistance [15, 16]. In fact, cancer cells take advantage of TME for their metabolism.

### The destructive effects of TME on immune cells

The TME is composed of heterogeneous cell populations and different molecules, with non-cancerous cells (stroma) including fibroblasts, immune and inflammatory cells, and endothelial cells [17]. Each of these cell types may have a different function within the TME, however, together they facilitate the tumor mass in gaining specific features, such as alteration of cellular phenotype and cancer stem cell generation, impairment of cell cycle checkpoints, immune evasion, angiogenesis, and tumor metastasis [8, 18]. Due to the defective vascular function and metabolic shift in TME, L-lactate, ketone bodies, and waste metabolites accumulate in the TME, providing a low PH environment that lends itself to a metabolic shift in immune cells, resulting in M2 tumor-associated macrophage (TAM) polarization, accumulation of myeloid-derived suppressor cells (MDSCs), and regulatory T cell differentiation [19], ultimately resulting in the inhibition of the anti-tumor function of T cells [20]. Moreover, lactate also significantly inhibits T cell expansion and stimulates the basis for their apoptosis [21]. Lactate directly has a massive impact on the anti-tumor response of CD8^+^T cells and natural killer (NK) cells [22, 23]. Tumor-derived lactate also affects macrophage polarization and low pH prevents the generation of lymphocyte-activated killer cells (LAKs) and cytokine production by T lymphocytes. Interestingly, it has been indicated that this condition is reversible and that T cell function becomes normal after a 24-h culture in lactate-free cell culture media [21, 24]. There is also nutrient competition between cancer and immune cells due to the high demand of both for energy, in which cancer cells conquer immune cells through their effective approaches in glucose consumption and ATP production [25, 26].

The secretome of CAFs includes different kinds of cytokines and chemokines, such as interleukin (IL)-6, TGF-β, and CXCL12 [27]. IL-6 and TGF-β serve as a CAF-autocrine self-activation loop, maintaining the the activation of CAFs [1]. Secretion of CXCL12 by CAFs results in an immunosuppressive TME through the recruiting of CD4^+^/CD25^+^ T cells and their differentiation into CD25^+^/ FOXP3^+^ regulatory T cells, which impacts the number of CD8^+^ T cells in the tumor site [28]. CAFs also significantly modulate the CD4^+^/CD25^−^ (effector T cell) proliferation rate by enhancing the capacity of regulatory T cells (Fig. 1) [6].

The impressive role of CAF-derived CXCL12 in breast cancer metastasis has also been shown in tumor invasion to the brain. Recent data have clarified that CAFs migrate to a new organ and then start to secret CXCL12, right at the beginning of tumor migration. Consequently, tumor cells expressing high levels of CXCR4 migrate toward a gradient of chemokine concentration to the new tissue [29]. This phenomenon has been confirmed by blocking the CXCR4/CXCL12 axis in some tumors [30, 31]. A study on the A549 lung cancer cell line demonstrated that CAFs enhance chemo-resistance in malignant cells by secreting CXCL12. The interaction of CXCL12 with CXCR4 on the surface of cancer cells upregulates the expression of Bcl-xL and inhibits apoptosis induced by chemotherapy [32]. CXCL12 also recruits bone marrow-derived endothelial precursor cells for angiogenesis and tumor metastasis [33]. IL-6 and TGF-β restrict the maturation of dendritic cells, with the result that these cells are probably no longer able to capture and present tumor antigens to T cells, ultimately resulting in T cells being anergic [34]. In addition, CAF-derived TGF-β decreases cytotoxic T cell differentiation, which brings about promotion of regulatory T cells in the tumor site [35]. Although the TME is not an appropriate environment for effector T cell differentiation, it provides a susceptible condition for regulatory T cell function due to the presence of some soluble factors, such as CCL2 and TGF-β, capable of suppressing the anti-tumor immune response [36]. Secretion of IDO (indole oxygenase) by CAFs resuls in the catalysis of a crucial amino acid, tryptophan, during T cell activation, which inhibits T cell clonal expansion. This enzyme is also involved in arginase metabolism, impairs aerobic glycolysis, and inhibits the expression of activation markers, such as CD25 and CD28, thereby limiting cellular proliferation and resulting in T cell apoptosis. By removing these important amino acids, the TME impairs T cell function [1, 36]. Due to the fact that they promote an immunosuppressive TME, CAFs are a serious obstacle to efficacious cancer immunotherapy [6]. Metformin, a widely used drug in the management of diabetes, with promising anti-cancer effects in terms of TME modulation and CAFs inhibition, may play a critical role in inhibiting cancer progression [37]. Moreover, downregulation of hypoxia-inducible factor-1 alpha (HIF-1α) expression in CAFs via metformin was found to decrease breast cancer cell invasion [38]. Also, metformin has been shown to inhibit CAF differentiation in the TME, which suggests a promising therapeutic approach in cancer therapy [39].

 In this review, we describe the metabolic cross-talk (metabolic symbiosis or cross-feeding) between tumor and CAFs and also highlight the main anti-tumor effects of metformin against cancer progression.

## Tumor progression via cross-feeding

Metabolic reprogramming of CAFs directly affects cancer cell viability via metabolic flux. Upregulation of glucose transporter (GLUT) 1–3 expression enables CAF cell to produce high amounts of lactate and transfer it via a lactate shuttle to anabolic cancer cells [40]. This symbiotic metabolic shuttle has also been reported in arginine metabolism in ovarian cancer, in which arginine is consumed in tumor cells and catabolized to citrulline to be transferred into adipose stromal cells, where it again converts to arginine and returns to cancer cells. The nitric oxide (NO) produced in this process inhibits oxidation phosphorylation (OXPHOS) in cancer cells and activates glycolysis [41].

Taken together, taking cross-feeding and metabolic shuttles into account could be beneficial for a more fundamental understanding of tumor biology, and may be a promising area for therapy based on metabolic stress in cancer cells.

### The Warburg effect

Under normal conditions (normoxia) normal cells prefer to use the OXPHOS pathway to generate approximately 36 molecules of ATP from pyruvate in the Krebs cycle inside mitochondria [42]. In hypoxia, on the other hand, anaerobic glycolysis results in the generation of lactate from pyruvate in a process known as the “Warburg effect” [43]. In the 1920s, Otto Warburg reported that the rate of glucose consumption and lactate production in tumors even at normal oxygen pressure is very high, leading to acidosis, i.e., the Warburg effect [44]. According to Warburg, acidosis may be the origin of cancer cell generation. Warburg also assumed that cancer cells have impaired mitochondria and, consequently, the oxidative metabolism is also impaired so the cancer cells are dependent on the glycolysis pathway for energy production [45]. Although the reason why proliferating cells prefer the Warburg effect is not fully understood, studies have shown that it is probably due to providing metabolites for the pentose phosphate pathway [46].

### The reverse Warburg effect and metabolic coupling

**Fig. 2:**
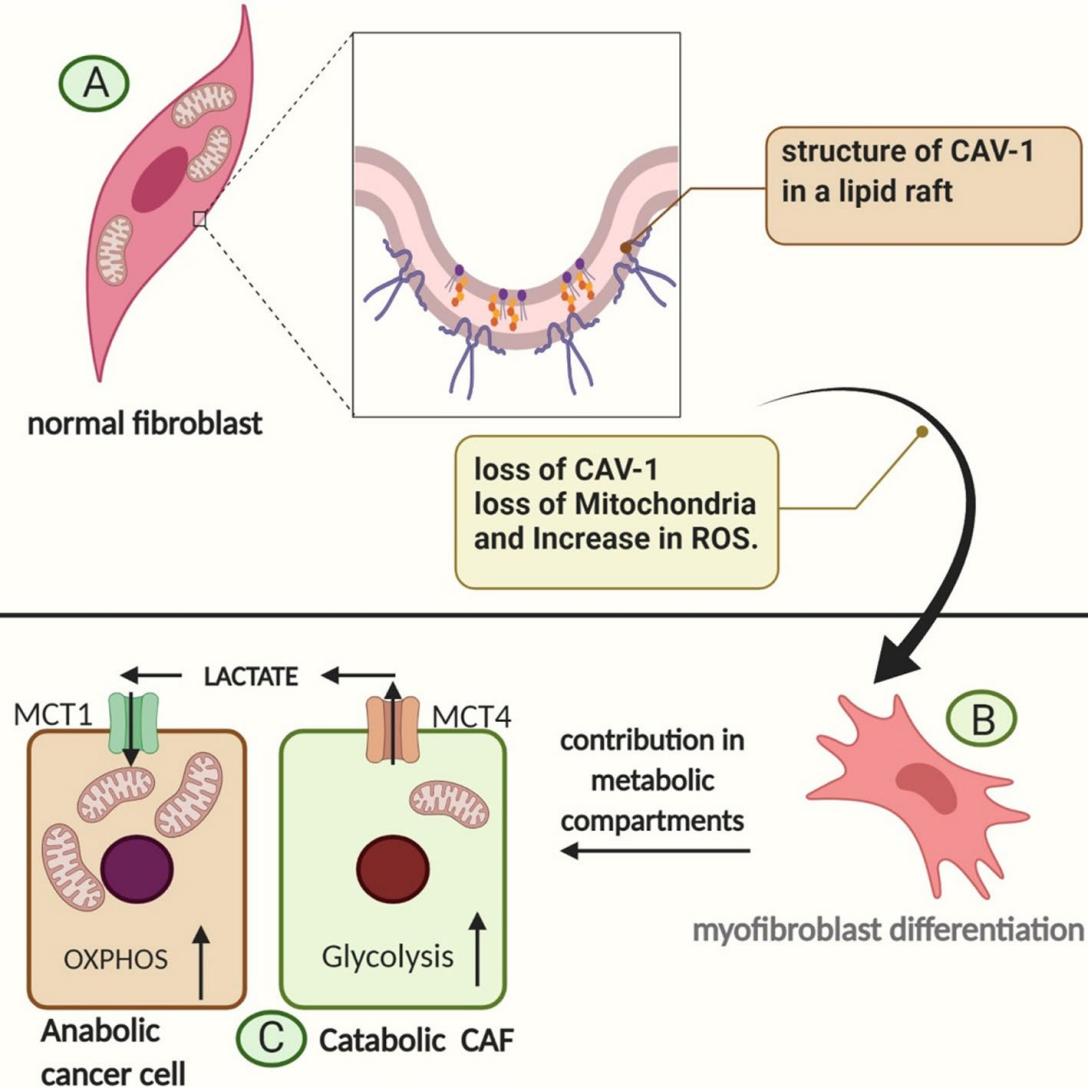
Loss of CAV-1 and myofibroblast differentiation. **a** In the cell membrane of normal fibroblasts, many CAV-1 molecules are located in the lipid raft. These cells mainly depend on the OXPHOS pathway for energy production due to the high number of mitochondria. **b** Exposure to ROS in the TME increases autophagy and loss of CAV-1. Lack of mitochondria shifts metabolism towards glycolysis, and lactate generation drives myofibroblast differentiation. **c** Catabolic CAFs tend to couple with anabolic cancer cells in a metabolic compartment. Anabolic cancer cells have mitochondrial reach cytoplasm and deeply depend on OXPHOS. This phenomenon is known as “the reverse Warburg effect.”* CAV* Caveolin,* MCT* monocarboxylate transporter,* OXPHOS* oxidation phosphorylation

While Otto Warburg’s experiments indicated that the rate of respiration in tumors iss low, the reverse Warburg effect has shown that respiration itself does not appear to be impaired [47]. In fact, the tricarboxylic acid (TCA) cycle in mitochondria is necessary for anabolic cancer cells because it enables proliferating cells to utilize intermediates of the TCA cycle [45]. The metabolic coupling of tumor and stroma in special structures called metabolic compartments revealed the secret of how the reverse Warburg effect serves to facility tumor viability [48]. Metabolic compartments consist of two or three types of cells in which one or two CAFs are physically joined to one cancer cell for feeding purposes; this process is also called metabolic coupling (Fig. 2) [49, 50].

How do these structures take shape? Cancer cells rewire the metabolism of surrounding cells in the TME [51]. Metabolic cross-talk between CAFs and tumor cells is very indispensable to tumors because it allows malignant cells to become more aggressive and be resistant to therapies [52]. Non-cancerous cells of stroma, such as vascular cells, immune cells, and fibroblasts, also lose their main function, ultimately to be exploited by tumor cells and to serve them in cancer progression and invasion [53]. Metabolic switches in both tumor and stroma are reprogrammed by malignant cells through the utilization of HIF-1α and ROS, such as H_2_O_2_, to induce oxidative stress, mutagenic evolution, and genomic instability in normal fibroblast cells, so that these cells obtain new metabolic phenotypes to become more glycolytic. [54]**.** Metabolic reprogramming in normal fibroblasts changes them into catabolic CAFs [43]. These cells prefer to provide lactate from glucose to feed cancer cells [55, 56]. To efficiently take up glucose, catabolic CAFs increase both glucose transporter 1 (Glut1) expression and a glycolytic enzyme called hexokinase (HK2) [57]. Consequently, L-lactate, pyruvate, and ketone bodies produced by catabolic CAFs feed the mitochondrial OXPHOS pathway in anabolic epithelial cancer cells, which is referred to as the “reverse Warburg effect” [24]. This phenomenon, which is also called “metabolic synergy,” mainly relies on catabolic CAFs [53]. Lisanti et al. studied the xerograph model of breast cancer and demonstrated that while lactate does not stimulate primary tumor generation, it could increase by approximately tenfold the risk of lung metastasis [58]. In the same study, these authors also evaluated ketone bodies and found that that 3-hydroxy-butyrate (a ketone body) could increase tumor viability by approximately 2.5-fold [58].

Tumor mass itself has a heterogeneous metabolic phenotype, a part of which depends on the glycolysis pathway while other parts rely on OXPHOS, in catabolic and anabolic cells, respectively [54]. Anabolic cancer cells have a mitochondrion-rich cytoplasm, and therefore it is expected that mitochondria in anabolic cancer cells serve as both a “powerhouse and Achilles heel” in tumor cells [25]. To evaluate this, stromal cells were transferred with cargos containing mitochondria in a study on the co-culture of stromal cells and mitochondrial-deficient lung cancer cells, with the results showing the importance of this organelle in anabolic cancer cells [59, 60]. In another study, the blockage of OXPHOS was found to limit drug resistance in lung cancer [61].

In metabolic coupling, catabolic CAFs directly transfer oncometabolites to cancer cells by an important channel in their membrane called the monocarboxylate Transporter (MCT4) which is also known as a catabolite exporter [62]. In contrast, anabolic cancer cells exemplify a membrane catabolite importer channel known as MCT1 (Fig. 2) [63]. These two channels are considered to be an ideal target for cancer therapy [54]. This entire process is hindered by MCT1 blockage, which demonstrates that lactic acid may use this channel in tumor cells and affect tumor cell signaling [64]. Furthermore, hypoxia and pseudohypoxic response make a metabolic shift toward glycolysis in the stroma and anti-apoptotic response in tumor cells via HIF-1α and the PI3K/AKT/mTOR axis [19]. In addition to playing a pivotal role in metabolic reprogramming, HIF-1α also potentiates metabolite shuttling by increasing the expression of MCT4 in CAFs [65].

## The role of caveoline-1 in cancer metabolism

One of the main causes of oxidative stress and metabolic shift in the TME is loss of caveoline-1 (CAV-1) [66]. The loss of CAV-1 through the increase in HIF-1α results in an upregulation of MCT4 expression due to the metabolic shift in CAFs towards catabolism [67]. CAV-1 is one of the three subtypes of caveolae (CAV-1, -2, -3) that are involved in lipid raft molecules. Lipid rafts are special intra-membrane components that are tightly packed and consist of saturated fatty acids, ceramide, sphingolipid, and cholesterol [68]. The main roles of lipid rafts are in cellular signal transduction, cholesterol transport, cytoskeletal organization, and vesicular transport (autophagy and endocytosis) [69]. CAV-1 is broadly involved in many tissues, CAV-2 exists where CAV-1 is expressed due to its dependency on CAV-1 for stabilization, and CAV-3 has limited distribution and is only expressed in the muscles [67]. CAV-1 interacts with some oncogenes (e.g., HRAS and SRC) and modulates their activities [70]. CAV-1 also binds to eNOS (epithelial NO synthase), resulting in the inhibition of this enzyme. Loss of CAV-1 leads to the activation of eNOS and NO production, which in turn leads to interactions with mitochondrial complex I and II, resulting in mitochondrial dysfunction and ROS generation [71]. Therefore, oxidative stress and loss of CAV-1 in CAFs lead to more CAV-1 fate in a positive feedback loop during autophagy in CAFs [72].

A study by Martinez et al. indicated that co-injection of CAFs and the CAV-1-deficient MDA-MB-231 cell line may increase the tumor mass by more than fourfold. The loss of CAV-1 has been shown to lead to an autophagic phenotype in CAFs, providing more energy-rich metabolites [73]. In that study, when mitochondrion-derived oxidative stress was decreased by SOD2 (superoxide dismutase 2), tumor progression caused by CAV-1-deficient CAFs was also decreased by twofold, thereby emphasizing the the role of mitochondrial oxidative stress [73]. Thus, loss of mitochondria results in a metabolic shift from OXPHOS to glycolysis, bringing about the generation of catabolic fibroblast and myofibroblast differentiation (Fig. 2) [67, 74].

In the xenograft model of breast cancer, loss of CAV-1 in stroma promoted tumor growth [73]. Loss of CAV-1 also occurs in some other types of cancer cells, such as liver carcinoma, T cell lymphoma, and colon cancer [75]. Unlike CAFs, loss of CAV-1 in cancer cells may lead to several consequences, such as OXPHOS pathway activation, mitochondria biogenesis and autophagy induction, apoptosis resistance, increased metabolite uptake through the expression of MCT1, and increased cell proliferation [76]. Indeed, when the CAV-1 is decreased, genes responsible for myofibroblast differentiation or oxidative stress-derived hypoxia are overexpressed. These genes encode > 40 proteins in myofibroblasts, some of which are collagen genes, genes upregulated in CAV-1 (−/−) cells, and gene sets related to the "reverse Warburg effect," such as glycolysis and HIF targets [73, 77]. Studies on patients with head and neck squamous cell carcinoma (HNSCC) reported that treatment with metformin increased CAV-1 levels and therefore may disrupt cancer metabolism, resulting in tumor apoptosis [78]. Increased levels of CAV-1 in breast cancer via metformin also improved the efficacy of trastuzumab emtansine (T-DM1), an antibody drug conjugate [79].

### What causes a decrease in CAV-1?

Studies have indicated that TGF-β is one of the factors driving the downregulation of CAV-1 [80]. TGF-β also promotes autophagy, mitophagy, and metabolic reprogramming of CAFs, resulting in tumor metastasis independent of angiogenesis [81].

Interestingly, in a positive feedback loop, the downregulation of CAV-1 intensifies TGF-β signaling pathway by inhibiting destruction of the TGF-β receptor [53]. Independent of the type of cells producing TGF-β, this cytokine influences adjacent cells and increases the level of myofibroblast markers [82]. TGF-β-derived tumor cells may not directly affect tumor growth, but rather activate fibroblast cells through a paracrine mode by downregulating CAV-1 and metabolic reprogramming the metabolism of these cells towards catabolic CAFs. Therefore, it indirectly facilitates tumor viability [83].

Moreover, the autocrine effects of TGF-β in metabolic alteration can be extended to stromal cells and may intensify tumorigenesis [84]. However, an increase in ROS caused by mitochondrial dysfunction is common in the TME due to upregulation of NADPH oxidase 1 (NOX-1), NOX-4, and modification of antioxidant enzymes, all of which occur before hypoxia imposes its effects [53].

Furthermore, glycolytic enzymes, such as pyruvate kinase (PKM2) and lactate dehydrogenase (LDH), increase upon the downregulation of CAV-1, which makes CAFs more efficient in glucose metabolism in synergy with the upregulation of GLUT3 [85]. Another main factor in the loss of CAV-1 is HIF-1α [86]. In hypoxia and when HIF-1α is overexpressed, CAV-1 level decreases directly, leading to a more glycolytic phenotype among CAFs, which is the main mechanism by which HIF-1α promotes tumor growth [67].

CAV-1 also enhances the mitochondrial functioning, and it has been suggested that its expression is increased in the mitochondrial membrane of cancer cells, but its expression should follow a threshold. Indeed, the overexpression of CAV-1 in cancer cells may increase mitochondrial-dependent apoptosis [75].

## Metformin and cancer therapy

Metformin (1, 1-dimethyl biguanide) is a cost-effective and safe medication that helps diabetic patients to reduce their blood sugar levels. It has exhibited anti-tumorigenic effects in both experimental and clinical studies, suggesting a promising potential role in the treatment of multiple cancer treatments, such as ovarian cancer, lung adenocarcinoma, and breast cancer [87–90]. Metformin has been widely used by diabetic patients for over 40 years, but more recently a growing body of evidence suggests that metformin may also modulate the phenotype and the number of non-transformed cell types in the TME, such as CAFs, endothelial cells, and innate and adaptive immunity cells, including TAMs and T-lymphocytes, by multiple strategies [91]. For example, metformin plays a significant role in the regulation of metabolic reprogramming in both cancer cells and tumor stroma [92]. Also, several groups of researchers have reported the effectiveness of metformin on fibroblasts and malignant cells at the same time via co-culture system (Table 2).

**Table 2:** Key effects of metformin on cancer-associated fibroblasts during cancer therapy

Mechanism/function of metformin	Results	Reference
Upregulation of calmodulin-like protein 3 (Calml3) in CAF isolated from gastric cancer tissues	Suppressed CAF-mediated proclonogenic effect on cancer cells	[178]
Inhibition of CAF-derived interleukin 6 secretion via suppressing of NF-κB signaling in The fibroblast cell line MRC5, and primary fibroblast of ovarian cancer specimen	Alleviation of stromal inflammation in ovarian cancer	[149]
Inhibition of cell growth and induction of apoptosis in OSCC cells by activation of the AMPK pathway, which is followed by downregulation of Bcl-2 expression, upregulation of the Bax/Bcl-2 ratio, and induction of cleaved PARP	Co-culture of NOFs with OSCC inhibited anti-tumor effects of metformin and protected tumor cells from apoptosis through inhibiting the activity of AMPK and PARP. Also, protection of mitochondrial memberane, indicating the supportive effect of fibroblasts in tumor progression	[179]
Interruption of HIF-1α-driven SDF-1 signaling in CAFs obtained from breast cancer tissues	Decreased breast cancer cell invasion	[38]

*AMPK* AMP-activated protein kinase, *NOFs* normal oral fibroblasts, *OSCC* Human oral squamous cell carcinoma line,* PARP* poly(ADP-ribose)polymerase-1

Zechner et al. [93] found that when metformin and gemcitabine were administered in combination to primary tumors, metformin affected cancer cells near the desmoplastic stroma more effectively, while gemcitabine inhibited the proliferation of those cells distant from the stroma. In addition, Hesler et al. [94] demonstrated that CAFs can serve as sources of CYR61 in co-culture models and contribute to chemoresistance by decreasing the level of nucleoside transporters involved in the uptake of gemcitabine by cells. In the following sections, metformin is discussed in the context of its potential therapeutic role, including inhibition of the mTOR (mammalian target of rapamycin) signaling pathway, which may play a pivotal role in downregulating cancer proliferation.

### The effect of metformin on oncogenic signaling pathways

**Fig. 3:**
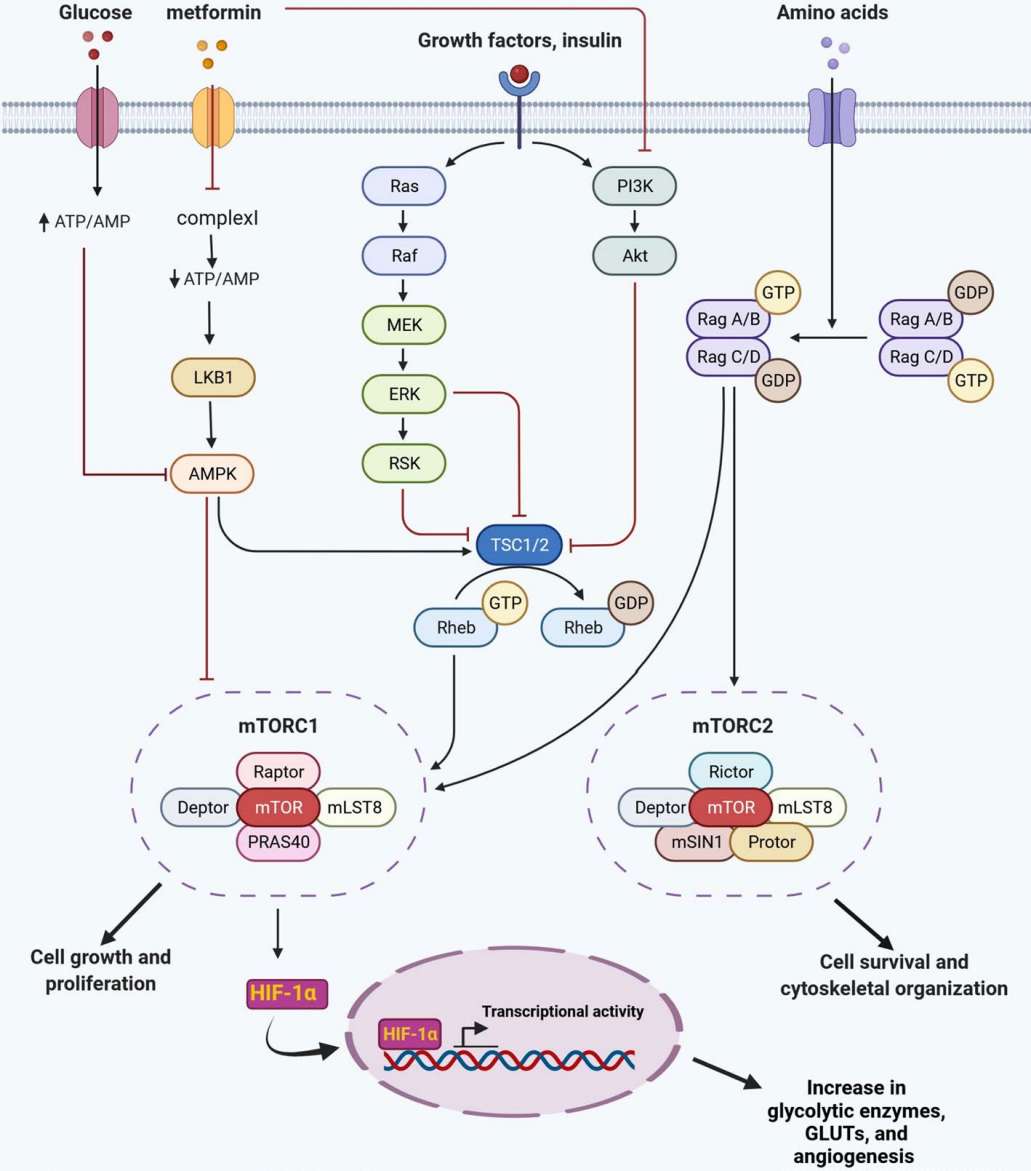
Metformin and oncosignaling pathways. The main anti-proliferative effect of metformin depends on inhibition of mTORC1. Inhibition of mitochondrial complex I by metformin and decreased ATP/AMP ratio activates the LKB1/AMPK pathway. AMPK is one of the main inhibitors of mTOR by activating TSC1/2 and downregulates mTORC1. However, the PI3K/AKT/mTOR axis is also inhibited by metformin (AMPK-independent manner). Although increased nutrients, such as amino acids and glucose, activate proliferation via mTOR activation, lack of metabolites inhibits cellular growth and division (not shown).* GDP* Guanosine-5′-diphosphate,* GLUT* glucose transporter, *GTP* Guanosine-5′-triphosphate, *mTOR* mammalian target of rapamycin

Metformin also targets ATP supply and energy homeostasis in cancer cells by inhibiting complex I in the mitochondrial electron transport chain, leading to ATP depletion and cell death [95]. When mitochondrial complex I is inhibited by metformin, both the NAD^+^/NADH ratio and glycolytic ATP production are decreased, leading to restriction of tumor progression (Fig. 3) [96]. It has also been demonstrated that metformin has inhibitory effects on cancer stem cell generation and, therefore, it may control tumor relapse [97].

### Stimulation of adenosine monophosphate-activated protein kinase

AMPK (adenosine monophosphate-activated protein kinase) is a pivotal metabolic sensor that is phosphorylated and activated by a serine-threonine kinase encoded by LKB1. LKB1 is a tumor suppressor molecule linked to the cellular metabolism and growth control pathway [98]. Moreover, an increased AMP/ATP ratio activates AMPK and LKB1, leading to phosphorylation of downstream substrates of AMPK, which in turn affects cellular metabolism and the dynamics of cell division during mitosis and inhibits cellular duplication (Fig. 3) [99]. Shi et al. [100] reported that molecular and pharmacologic knockdown of AMPK disrupts the growth inhibition effect of metformin in lymphoma cells, revealing that the main anti-proliferative effect of metformin may depend on AMPK activity. Metformin also activates autophagy in human hepatocellular carcinoma cells through the AMPK signaling pathway [101].

### Inhibition of the PI3K/AKT/mTOR signaling pathway by metformin

Cell growth and metabolism is regulated by mTOR, a serine/threonine kinase. The activation of mTOR stimulates anabolism, such as ribosome biogenesis and protein, nucleotide, fatty acid and lipid synthesis. Autophagy, however, is hindered by mTOR [102]. Conversely, inhibition of mTOR by metformin may activate autophagy [101]. Gain of function (GOF) mutations in upstream regulators of mTOR, such as PI3K, may directly activate mTOR, which has been reported in many tumors [103]. The loss of function (LOF) mutation of tumor suppressor PTEN results in mTOR activation [104].

Interestingly, oxidized ATM (ataxia-telangiectasia mutated protein kinase) upregulates the expression of PKM2 (pyruvate kinase M2) via PI3K/AKT signaling. PKM2 promotes both ATP generation in the glycolytic pathway and pyruvate production. Accordingly, PKM2 expression contributes to aerobic glycolysis of cancer cells for tumor cell growth and cancer progression [105]. Kexin Sun et al. [105] used KU60019 to inhibit oxidized ATM activation and observed that the result was a decrease in the levels of PKM2 proteins and p-AKT. They also found that the levels of PKM2 and p-AKT proteins decreased subsequent to ATM knockdown in hypoxic CAFs, showing that PKM2 expression is regulated by oxidized ATM and its downstream PI3K/AKT signaling. Hence, this study [105] demonstrates that in response to hypoxia, a high level of PKM2 in CAFs is critical for glycolytic pathway changes.

In addition to being affected by mutations, mTOR may also be modulated by nutrient sensing (Fig. 3), with a lack of nutrients via activation of the AMPK pathway inhibiting mTOR. On the other hand, mTOR regulates various metabolic pathways, including fatty acid, nucleotide, glucose, and amino acid pathways [106]. Metformin induces LKB1 and alternatively blocks mTOR by activating AMPK. In addition, overexpression of IGF-1R ( insulin-like growth factor 1 receptor) may contribute to cancer initiation via PI3K/AKT/mTOR signaling (Fig. 3). Metformin, however, by downregulating IGF-1 (insulin-like growth factor-1) diminishes tumor growth; as such in tumors with a high level of insulin, metformin may be a promising drug [107]. The inhibition of the PI3K pathway by metformin is also considered to be an important antineoplastic mechanism of metformin [108]. Inhibition of mTOR in metformin-treated breast carcinoma cells also downregulated HER2 (human epidermal growth factor receptor-2 HER2), in an AMPK-independent manner [109].

Tuberous sclerosis complex 1 (TSC1) and 2 (TSC2) are tumor suppressor factors that inhibit mTORC1 signaling (Fig. 3). Such inhibition of mTOR signaling, by downregulating molecules associated with protein synthesis in the ribosome, inhibits global protein synthesis [110]. Although, these signaling pathways may also be activated in normal cells, the difference is that during cancer progression these signaling networks, such as PI3K/AKT/mTOR, are highly activated without or with less dependency on extrinsic stimulators, such as growth factors [111].

### Nuclear factor kappa B pathway inhibition by metformin

Nuclear factor kappa B (NF-κB) is an important target in cancer therapy due to its important role in cancer proliferation and metastasis. Mutations in upstream NF-κB factors may activate the NF-κB signaling pathways to induce promoters of oncogenes to form a positive-feedback loop [112]. In their study on head and neck cancer (HNC), Qin et al. [113] found that IL-6 secreted by CAFs is the principal upstream molecule involved in the production of neoplastic osteopontin (OPN). Accordingly, during the interaction of CAFs with cancer cells, CAF-derived IL-6 via stimulation of the osteopontin-NF-κB signaling pathway promoted cancer cell growth, migration, and invasion [113]. Metformin, by upregulation of NF-κB inhibitor alpha (IκBa), inhibits NF-κB signaling and also attenuates tumor proliferation in MCF7 breast cancer cell line [114]. Other studies have also indicated that metformin diminished IL-6- and NF-κB- mediated epithelial-mesenchymal transition (EMT), one of the main factors in cancer metastasis [115].

### HIF-1α inhibition by metformin

Overexpression of HIF-1α has been demonstrated to play a significant role in the development and progression of multiple cancers [116–118]. In a recent study by Shao et al. [38], the effect of metformin on HIF-1α in the breast cancer TME was assessed. The results of western blotting showed increased HIF-1α expression by CAFs when co-cultured with breast cancer cells (MDA-MB-231 and MCF-7); metformin was shown to be able to attenuate the expression of HIF-1α in these CAFs [38]. The authors suggested that metformin could be used as a promising drug to target tumor-promoting signal transduction between CAFs and the breast cancer TME. In another study, metformin decreased the amount of HIF-1α accumulated by suppressing mitochondrial oxygen consumption and increasing cellular oxygenation, suggesting that metformin acts as an anti-cancer drug against hepatocellular carcinoma (HCC) [119]. Additionally, the author of a study on breast cancer reported that metformin treatment resulted in a decrease in the number of HIF-1α nucleus-positive cells in 4T1 tumors, together with a decrease in microvessel density. Taken together, these data provide important insights into the mechanism responsible for the metformin-induced inhibition of tumor angiogenesis and point to the possibility of HER2-induced angiogenesis being mediated by HIF-1α-VEGF (vascular endothelial growth factor) signaling [120]. Interestingly, HIF-1α increases LDH activity and HK2, promoting glycolysis, thus facilitating cancer progression [121]. Furthermore, TGF-β1 and PDGF derived from cancer cells through activation of the HIF-1α signaling pathway promote glycolysis in fibroblasts, resulting in CAF differentiation. Moreover, lysophosphatidic acid (LPA), a lipid mediator secreted by tumor cells, also activates HIF-1α signaling and glycolysis reprogramming in CAFs [122]. Therefore, hypoxia, as a main hallmark of cancer by shifting the metabolism from OXPHOS to glycolysis, plays a pivotal role in CAF generation. Hence, it seems that metformin by targeting HIF-1α could be used as a promising drug for cancer therapy to suppress the development and progression of multiple cancers.

### ERK signaling pathway inhibition by metformin

Recent studies have shown that a combination of metformin and cisplatin can synergistically suppress tumorigenicity in vitro and in vivo, inhibit the proliferation of gallbladder cancer cells, induce cell apoptosis, and arrest G0/G1 phase cell growth [123]. In addition, Kim et al. [124] found that the combination therapeutic regimen of metformin + BEZ235 (an inhibitor of the PI3K/AKT/mTOR pathway) synergistically promotes anti-tumor effects in HCT15 colorectal cancer cells via suppression of ERK phosphorylation. Further, it has been revealed that the GPER/EGFR/ERK signaling pathway increases the expression of β1-integrin and activates downstream kinases, which contributes to CAF-induced cell migration and EMT in tamoxifen-resistant breast cancer cells [125].

Recently, the MAPK/ERK pathway has been identified as a key mediator of the Warburg effect in both tumorigenesis and normal cellular functions. In cancer cells, induction of transcriptional regulators of glycolysis, the TCA cycle, and the aerobic glycolytic phenotype occur through the ERK1/2 pathway [126]. By activating the PI3K/AKT, MEK/ERK, and Wnt/β-catenin signaling pathways, oxidized ATM plays a critical role in the induction of the proliferation and glycolytic activity of breast CAFs [105].

However, the inhibitory effects of metformin on CAFs by targeting ERK pathways and related metabolic reprogramming are not well-understood. We suggest that future studies are needed to evaluate the possible inhibitory effects of metformin on ERK pathways in CAFs.

## Oncometabolites in cancer

Metabolites modulate the biological activity of cancer cells by altering signaling pathways. These signaling pathways may involve epigenetic modification, signal transduction, and intercellular communication, all of which are affected by oncometabolites [127].

Lactate is a major source of engery and long been regarded as an important molecules, and it has recently being classified as an oncometabolite. In vitro studies on this molecule indicate that the intrinsic and extrinsic presence of lactate activates the main oncogenes (MYC, RAS, and PI3KCA), transcription factors (HIF-1α and E2F1), and tumor suppressors (BRCA1, BRCA2). Additionally, cell cycle and proliferation genes involved in breast cancer (AKT1, ATM, CCND1, CDK4, CDKN1A, CDK2B) were also elevated in this study [128]. Lactate and succinate, two intermediates of the Krebs cycle, are induced by hypoxia. Together, these metabolites activate TAMs (tumor-associated macrophages) to help cancer to form new vessels [129]. An increase in the lactate level is sensed by NDRG3 (N-Myc downstream-regulated 3 NDRG3), which initiates the hypoxic response and angiogenesis via Raf-ERK1/2 signaling [130]. Oncometabolites involved in lipid metabolism also activate NF-κB and enhance genes of proliferation and angiogenesis in the mouse model of breast cancer [131]. Some of the oncometabolites are produced by mutant enzymes in cancer cells. The main example is 2HG (2-hydroxyglutarate)), which is produced by mutant isocitrate dehydrogenase 1 and 2 (IDH1/2) [132]. Mutated IDH1/2 can initiate a malignancy and has been reported in several cancers, such as myeloid leukemia, chondrosarcoma, and glioma [133]. 2HG modifies metabolic enzymes like ATP synthase and plays a role in metabolic reprogramming [134]. Furthermore, 2HG is a structural analog of α-ketoglutarate (α-KG) and modulates the TCA in the mitochondria. Its function as an oncometabolites was established by involving the inhibition of alpha-ketoglutarate-dependent dioxygenases [135]. It has also been shown that 2HG reshapes the metabolism of T cells and affects their polarization [136]. 2HG also affects tumor-infiltrated lymphocytes by activating STAT1 signaling and the production of CXCL10 [137]. Moreover, 2HG interferes with HIF-1α in T cell metabolism and the frequency of regulatory T cells. An increase in mTOR by 2HG has also been confirmed, which in turn enhances HIF-1α signaling [132].

### Metformin inhibits 2HG

The effects of metformin on oncometabolites have only recently been studied and, consequently, the number of studies on these effects is limited. However, a recent study by Oh et al. [138] on MCF7 breast cancer cell line and epithelial cell line MCF10A showed that upon metformin treatment, 2HG decreased by about 70%. Since the effect of 2HG is due to histone modifications, these findings indicate that histone epigenetic changes as a result of metformin treatment may decrease 2HG and tumor proliferation. Likewise, metformin-fed mice also exhibited significantly lower tumor volumes [138]. Another study found that IDH1-mutant cells are more sensitive to mitochondrial poisons like metformin, which has been confirmed by assays of cell proliferation, clonogenic potential, and mammosphere formation [139].

## Metformin and modulation of the immune system

Blocking mTOR in T-cells by metformin may shift the anabolic state to the catabolic phase and, consequently, they may differentiate from effector to memory, which may improve anti-tumor immune response in the TME [140]. T cells in the TME are found to have many metabolic limitations caused by immune checkpoints like programmed cell death protein 1 (PD-1) signaling. In brief, when PD-1 in effecter T lymphocytes interacts with PD-1L (PD-1 ligand) on the tumor surface, its signaling in T cells results in downregulation of GLUT-1 and glycolysis inhibition, exhausting the cells, while reverse signaling of PD-L1 increasing GLUT-1 and glucose uptake in tumor cells [141].

These findings indicate that working on immune checkpoint inhibitors due to their close cross-talk with T cell metabolism may be a promising therapeutic approach in boosting anti-tumor immunity [36]. A study on PD-L1 expression in non-small cell lung cancer (NSCLC) revealed that as the expression of PD-L1 in cells is increased, the expression of HK2, lactate production, and the rate of extracellular acidification in TME are also elevated [142]. Moreover, metformin has been shown to protect T cells from exhaustion and apoptosis caused by PD-L1 expression through endoplasmic reticulum-associated degradation (ERAD) of PD-L1 in cancer cells, which is an important issue in many tumors [143].

Metformin has been proven to improve the advantage of PD-L1 blockade by rescuing T cells from hypoxia through the inhibition of tumor cells from oxygen consumption, which greatly improves immunotherapy results. By the same token, the more oxidative metabolism that occurs in the tumor, the greater the exhaustion in T cells [144]. Transcriptome sequencing studies have revealed that metformin affects immune cell function by altering the whole-blood transcriptome profiles of healthy individuals. This same study also indicated that metformin exerted its effect by improving the intestinal immune system [145].

Although alteration to the intestinal microbiome and, consequently, to the immune system response are not the main purposes of metformin, it may directly activate the innate immune system through the MAPK pathway [146]. The immunosuppressive TME caused by cytokines produced by cells, such as MDSCs and regulatory T cells, may lead to failure in immunotherapy of breast cancer. Studies on naïve CD4^+^CD25−FOXP3−T cells have shown that metformin decreased iTreg differentiation even in the presence of TGF-β. Furthermore, Foxp3 has been shown to be downregulated by metformin in these cells.

T cells have probably undergone metabolic reprogramming, which makes a metabolic shift to glycolysis instead of OXPHOS. Therefore, the production of both IL-10 and CTLA-4 is decreased [147]. The immunosuppressive effects of MDSCs and their accumulation in tumor sites has been shown to be inhibited by metformin [148].

Also, metformin, by downregulating NF-κB signaling and IL-6 production in CAFs significantly inhibits tumor growth in ovarian cancer. [149]. Moreover, in a study in which biopsy samples and surgery samples of patients suffering from HNSCC were compared, significant differences in the level of CAV-1 but not MCT4 were found in those patients who were taking high-dose (200 mg) metformin [78]. This result demonstrates that the effect of metformin on MCT4 in lung cancer needs to be investigated more thoroughly.

Studies on prostate and gastric cancer have also shown that metformin treatment decreases CXCL12/CXCR4 signaling, the main axis in tumor metastasis [150, 151]. Genetic studies, however, have shown that via some histone modifications, netformin re-sensitized multidrug resistance (MDR) breast cancer cells to therapy [152]. The efficacy and effectiveness of metformin have been indicated in immunotherapy [153], chemotherapy [154], and also in combination with other herbal drugs such as curcumin [155] (Table 3).

**Table 3:** Summary of clinical trials on metformin administration in cancer therapy

Title and identification	Intervention/treatment	Results
Phase II study of metformin for reduction of obesity-associated breast cancer risk [172, 173] NCT02028221	Metformin/placebo Metformin 850 mg 1 tablet by the oral route, then metformin 850 mg 1 tablet twice daily for the duration of the intervention period	Decrease in tumor size within 6–12 months, as well as serum level of insulin-like growth factor and serum insulin levels
Castration compared to castration plus metformin as first-line treatment for patients with advanced prostate cancer [174] ClinicalTrials.gov Identifier: NCT01620593	Metformin/placebo 500 mg metformin three times a day	PSA ≤ 4 ng/ml or undetectable value at 7 months Metformin did not affect metabolic syndrome followed by androgen deprivation therapy (ADT)
A Trial of standard chemotherapy with metformin (vs placebo) in women with metastatic breast cancer [175] ClinicalTrials.gov Identifier: NCT01310231	Metformin 850 mg in addition to standard chemotherapy (including anthracyclines, platinum, taxanes or capecitabine; first or second line)	Progression-free survival was not affected; overall survival, and response rate in non diabetic breast cancer patients
Evaluation of metformin, targeting cancer stem cells for prevention of relapse in gynecologic patients [88] ClinicalTrials.gov Identifier: NCT01579812	Receiving metformin prior to primary surgery. After surgery patients received metformin prior to the initiation of chemotherapy	Improved overall survival in ovarian cancer patients, and by epigenetic changes in the tumor stroma may resensitize cancer cells to chemotherapy
Bicalutamide with or without metformin for biochemical recurrence in overweight or obese prostate cancer Patients (BIMET-1) [176] ClinicalTrials.gov Identifier: NCT02614859	Metformin 1000 mg Bicalutamide 50 mg daily	Metformin monotherapy had better results in decreasing PSA compared with combination therapy. Also, combination therapy and monotherapy both improved anti-tumor activity of T cells and NK cells and. therefore, modulated immune system

*NK* Natural killer, *PSA* prostate-specific antigen

The effects on metformin on tumor-infiltrated T-cells (TILs) also indicates that this herbal-based drug can reduce exhausted CD8^+^ T cells [156]. The results of a study on HER-2-positive metastatic breast cancer cells indicated that due to the fundamental effects of CAV-1 on endocytosis, pretreatment with metformin increased CAV-1-mediated drug internalization into malignant cells, leading to a decrease in malignant cell viability [79]. Based on the critical role of CAV-1 in AMPK activation and homeostasis of energy, a study on cell lines of non-small-cell lung cancer (NSCLC) revealed that the expression of CAV-1 seems to be necessary for a higher efficacy of metformin via the increase in AMP/ATP ratio and AMP phosphorylation [157].

## Metformin blocks the metabolic cross-talk of tumor and CAFs

Although cancer metabolism is an old subject, it has been recently been taken into consideration as a promising therapeutic approach. As mentioned earlier, disruption of oxidative phosphorylation and metabolic stress caused by inhibition of ATP production in mitochondria is a notable anti-neoplastic effect of metformin [96]. Indeed, studies on the metabolomics of human tumors and mitochondria indicated that metformin affects several metabolic pathways in mitochondria, such as the purine, pyrimidine, and glutathione metabolism pathways [158].

Studies on cancer metabolism, however, have revealed that metformin may target metabolic cross-talk between the tumor and stroma via blocking of the lactate flux channel MCT4, which is highly expressed in catabolic CAFs [99], resulting in these cells being no longer able to transfer the final products of glycolysis to malignant cells. This blockage imposed metabolic shock in the tumor cells, leading to apoptosis [54, 96, 159]. Attenuating lactic acid excision followed by MCT4 blocking has shown that metformin may downregulate acidification of the TME in favor of the immune response [24]. Studies have proven that metabolic reprogramming, caused by oxidative stress, has been inhibited by the antioxidant effect of metformin [160, 161].

Metformin also directly downregulates the glycolysis enzyme HK2, which impairs tumor cell growth [162]. Metabolic compartments made by catabolic CAFs and anabolic cancer cells may also impair tumor cell growth because CAFs are highly glycolytic and addicted to glucose to provide energy for cancer cells; inhibition of glycolysis by metformin results in cancer cells with a deep metabolic stress [163]. Cancer cells also absorb fatty acids from the TME, utilizing these in cell signaling, building blocks for membranes, and fuel to thrive [164]. Fatty acid synthase is also inhibited by metformin, and a shift in cholesterol and lipid metabolism may deprive cancer growth and proliferation [165, 166]. A reduction in insulin resistance and downregulation of the number of membrane GLUTs are also induced by this drug [167]. Moreover, LDH, which is the main enzyme in lactate generation and TME acidosis, is inhibited by metformin, which may result in modulation of the pH of the TME [168].

Acidity is an important feature of the TME and associated with a poor prognosis, which hinders most therapies [169]. In this regard modulation of the pH of the TME is considered to be a promising therapeutic approach [170]. Although the efficacy of metformin has been shown to be reduced during cellular apoptosis and the metabolic process in high glucose media [171], this drug has effective anti-cancer mechanisms that remain to be more evaluated.

## Conclusion

The TME is a complicated condition with different types of cells and molecules which are directed towards facilitating tumor progression. Metabolic interactions between the tumor and stroma solve the problem of the high demand of malignant cells, providing an energy source. Moreover, metabolic compartments made by catabolic CAFs and anabolic cancer cells also provide high-energy metabolites for tumor progression. Metformin, however, could be a promising anti-cancer adjuvant, which could be used in addition to first-line cancer treatments. Since this promising drug targets the cellular ATP pool to impose metabolic stress, it may provide novel ways to target non-stop growth and therefore it should be studied further. Cancer metabolism and metabolic cross-talk in TME, as well as metabolic adaptation, seems to be the Achilles heel of cancer growth, which could be a potential therapeutic approach, and needs to be investigated thoroughly. Also, it seems that the supportive role of CAFs in tumor progression should be considered when new drugs are being designed due to their destructive role in promoting drug resistance and tumor metastasis.

## Data Availability

Not applicable.

## Abbreviations

CAFs: Cancer-associated fibroblasts
CAV: Caveolin
EMT: Epithelial-mesenchymal transition
HIF-1α: Hypoxia-inducible factor-1 alpha
HK: Hexokinase
MCT: Monocarboxylate transporter
MDSCs: Myeloid-derived suppressor cells
MSC: Mesenchymal stem cells
NSCLC: Non-small-cell lung cancer
OXPHOS: Oxidation phosphorylation
ROS: Reactive oxygen species
TAM: Tumor-associated macrophage
TCA: Tricarboxylic acid cycle
TIL: Tumor-infiltrated lymphocytes
TME: Tumor microenvironment
